# Epidemiology in dialogues: belonging to the Collective Health field, practices and theoretical tensions

**DOI:** 10.1590/1980-549720250002.supl.1

**Published:** 2025-11-28

**Authors:** Rita Barradas Barata

**Affiliations:** I Faculdade de Ciências Médicas da Santa Casa de São Paulo – São Paulo (SP), Brazil.

**Keywords:** Epidemiology, Public health, Philosophy

## Abstract

**Objective:**

In this essay, we discuss the belonging of Epidemiology to the Collective Health field, theories and contributions of Epidemiology to the health work process and to the impact assessment of Collective Health programs and actions, and the practical and theoretical tensions in the discipline and in the field.

**Methods:**

The essay was conducted based on the theoretical contributions of various authors who reflected on Epidemiology throughout the 20th and early 21st centuries as well as the author's own experiences. It is organized into four thematic sections: the discipline's role within the field of Collective Health; the relations between Epidemiology and health practices; practical and theoretical tensions within the discipline itself and other disciplines within the field; and reflections on possible dialogues.

**Results:**

We suggest resorting to transdisciplinarity, understanding the dynamics of scientific fields, overcoming false hierarchies between types of knowledge, defeating disciplinary limits, and decentralizing network connections as ways of coping with and overcoming difficulties.

**Conclusions:**

The reward for this effort lies in the possibility of achieving effective responses to complex issues that, in addition to improving the health situation of populations, can be guided by the moral imperative of reducing inequalities and social injustices, respecting human rights, and building a more supportive and healthy society.

## INTRODUCTION

Epidemiology is one of the key disciplines of the field of Collective Health linked to the understanding of the population dimension of the health-disease process both in the study on the distributions of health statuses and events and in the elucidation of the relations between epidemiological profiles and social, cultural, and political determinants, at each stage of the historical movement of society.

As a discipline dedicated to strategic research in the field of health, the produced knowledge has implications in the practices of prevention and control of diseases and harms, maintenance of health status, formulation of action strategies, and impact assessment.

The tensions within the discipline stem from different theoretical and methodological perspectives and, within the field of Collective Health, from the struggle for financial, political, and symbolic capitals existing in any scientific field.

In this essay, we aim to discuss these points and propose some forms of dialogue to overcome internal and external tensions.

## METHODS

The reasoning presented stem from the author's reflections, with the aid of the selection of books and articles by different authors who reflected on Science and Epidemiology throughout the 20th and early 21st centuries, to elaborate a coherent and logical narrative addressing the different aspects indicated in the title.

The essay is organized into four thematic sections: the belonging of Epidemiology to the field of Collective Health; the relations between Epidemiology and health practices; the theoretical and practical tensions within the discipline itself and in the field of Collective Health; and the possible dialogues in the discipline itself and with others in the same field.

## RESULTS

### Epidemiology's belonging to the field of Collective Health

According to Alfredo Morabia^1^, Epidemiology was born in 1662, in England, when John Graunt began to systematically analyze the lists of causes of death. The origins of the discipline depended on the formulation of the concept of population and the comparison between groups, differentiated by demographic, economic, social, cultural, and political characteristics for the study of population distributions of diseases, health issues, and health statuses and the identification of the determinants of these occurrences.

As population thinking is not intuitive, it has taken time for the discipline to be constituted as such, although notions about health and hygiene, as well as about diseases and medicine, were historically older.

The mortality lists of parishes in London, England, were one of the first systematic records that allowed the surveillance of plague cases to guide the actions of health committees in each neighborhood. In 1662, John Graunt published the book *Natural and Political Observations Made Upon the Bills of Mortality,* analyzing data from the previous 50 years, constituting a milestone for the development of epidemiological knowledge.

The concept of population health emerged only at the end of the 18th century, associated with the Enlightenment philosophical thinking that adopted as a principle the idea that protection against diseases should be extended to all citizens and hoped that advances in science and technology would enable epidemics to be prevented and the health of the population to be improved^2^.

The development of epidemiological knowledge and the constitution of the scientific discipline took place in the 19th century, simultaneously with public health reforms in European countries and the movement of social medicine. Since the beginning, the Public Health movement has been in conflict with dominant economic interests, demonstrating the ambivalence between the benefits that economic growth could bring to the health of the population and the restriction of public health actions imposed by the ruling classes^2^.

Historically speaking, all transition periods between modes of production showed population growth accompanied by greater susceptibility to diseases and relative worsening of population health. The accumulation of wealth alone does not guarantee health benefits, and social and political negotiations are necessary to guide the distribution of goods aimed at the majority of the population. Historically, economic growth has been intrinsically disruptive, in the absence of national and local policies aimed at avoiding or minimizing the so-called four Ds: disruption, deprivation, diseases, and deaths.

In the last decades of the 20th century, the process of concentration of wealth occurred in an accelerated way, while social welfare policies implemented in rich countries and, to some extent, in middle-income countries began to be dismantled, deepening the negative effects of inequalities on the distribution of wealth, on living and health conditions around the world.

Epidemiology is the basis for understanding and changing the social patterns of distribution of health, disease, and well-being, and should produce theories to explain the processes that shape epidemiological profiles in the population, understand the determinants of social inequalities, and guide the formulation of public policies for coping with it. The first duty of an epidemiologist is to measure and disclose the damage to population health resulting from economic growth and the concentration of wealth, to mobilize political forces and public opinion^3^.

Depending on the theory about the health-disease-care process, the understanding about the problems will be individual- (lifestyle theory, clinical epidemiology) or population-based (social production of health and disease), and, consequently, the intervention proposals will be different.

Collective Health emerged in Brazil as a movement with historical roots in social medicine, as opposed to preventive medicine and conventional Public Health. Conceptualizing it as a field facilitates the understanding of the multiplicity and co-occurrence of knowledges and practices, constituted around the health-disease-care object in the collectivity, as an expression of the process of social organization^4^.

The field of Collective Health is structured in three axes of knowledges and practices in the collective dimension: Epidemiology, Social Sciences in health, and Planning, organization and management of health policies, programs, and services^5^. Therefore, more than belonging to the field of Collective Health, Epidemiology is a key constituent of this field, along with other types of knowledge that structure it.

### Epidemiology and health care

Different definitions of Epidemiology, including that of the Dictionary of Epidemiology^6^, denote the double demand for knowledge production and its application to solve health issues.

To understand the role of Epidemiology in health practices, it is worth considering the particularities of the work in Collective Health and its primary purpose, which is to modify population health conditions^7,8^.

The object of the health work is the *normativity of the ways of walking through life* of human groups. The essential capacity of living beings mobilized by health is the ability to recognize everyday challenges and respond to them in a normative way, creating new norms in the face of difficulties^7,8^.

Health agents and professionals, in their work, operate types of knowledge based on life sciences and professional practice, combining intuition, empathy, scientific knowledge, and material and immaterial techniques, developing technologies and action models. The technological arrangements articulate the social needs in health to a certain technical rationality (available knowledge and instruments) and forms of organization, aiming at the production of a good capable of meeting health needs^7,8^.

Epidemiological knowledge and practices play several roles in technological arrangements in health services guided by the concept of Collective Health and population focus. Among them, we mention the understanding of the distribution of health, disease, and well-being in the different population groups; the identification of the processes that shape these distributions; more effective ways of addressing these processes to impact distributions in order to improve the health status; and evaluation of available techniques and technologies and interventions^9^.

In particular, regarding the implementation and evaluation of the impacts of health programs and policies, the inverse equity hypothesis developed by Cesar Victora et al. broadens the understanding of the ability of collective health interventions to reduce or expand social inequalities, depending on the model of implementation adopted and the prior consideration of inequalities that modify or modulate the effects of interventions^10^.

The inverse equity hypothesis postulates that new interventions initially reach the social layers with higher socioeconomic level and that, due to their living conditions, share situations that favor the incorporation of actions and services, and later these innovations may or may not be incorporated by the poorest layers, depending on the level of population coverage achieved by the interventions^10^.

Furthermore, depending on the implementation strategies used, new programs and policies may achieve high coverage in the layers that concentrate power and wealth, with favorable effects concentrated at the top of the social pyramid and distancing from the other layers, increasing inequalities. It is also possible that proposals of more universal scope reach several social layers with different positions of power and wealth in a similar way, abandoning the poorest, with an increase in inequality unfavorable to those whose social insertion is more precarious. Likewise, we can also identify situations of a gradient of coverage inversely proportional to the socioeconomic position of the different class fractions. Therefore, it is very important that the formulation of implementation strategies consider the existence of inequalities and the characteristics of different social fractions to prevent health practices from being themselves amplifiers of social inequalities in health^11^.

Thus, Epidemiology, besides being key to the structuring of the scientific field of Collective Health, is presented as an important discipline for health practices, providing relevant knowledge for the organization of the health work process in the population dimension, guiding the formulation of programs and policies based on scientific evidence, and assisting in the evaluation of the impacts of interventions.

### Theoretical and practical tensions in the discipline and in the field

Tensions within the discipline itself have been discussed in the last 25 years around the crisis of explanatory paradigms, the little attention given to theories of social determination, the importance given to methods and techniques of analysis aimed at etiological elucidation at the expense of relevant analyses for praxis in Collective Health, and the incipient development of the concept of health and well-being^12–14^.

The theoretical impoverishment of the discipline and the predominance of issues concerning methods and techniques of analysis have consequences not only for the explanatory power of the produced knowledge, but also for the practices in the field, as the focus on elucidation of etiological mechanisms has weakened the perspective of applying knowledge to solve problems in the population dimension^12,14^.

Krieger^14^ highlights the permanence of opposition between theories based on the so-called "lifestyle" and theories of social determination of the health-disease process. The author points out some divergent trends observed in the development of Social Epidemiology: on the one hand, the depoliticization of social determinants of health; on the other hand, the renewed emphasis on the political economy of health associated with the discussion of partisan, commercial, and social determinants of health, the increase in the visibility of Collective Health and Latin American social medicine, theories about health and human rights, population health and others.

Despite the multiple theoretical developments about the distribution and determinants of health and disease, the author points out the little attention given to the issues of distribution of health determinants, exposures and outcomes. Werneck^13^ highlights the extensive production in Epidemiology in the 2001–2024 period and "glaring absences" such as: gender and racism issues, populations in situations of social vulnerability, populations deprived of freedom, people with disabilities, human rights and health, preparation and response to public health emergencies, climate crisis, disinformation, intersectionality, among others. To these absences, we can also add the political polarization and its consequences on health and the new obscurantism and the growing social discredit of science as relevant historical and social processes at the beginning of the 21st century.

All these tensions, dilemmas, and theoretical gaps are reflected in the field of practices informed by epidemiological knowledge. The scarcity of the population perspective and the little importance attributed to the knowledge and understanding of distributions in the organization of services and in the process of health work reduce the effectiveness of policies, programs, and actions.

The lack of preparation of health professionals, with scarce training in Epidemiology and difficulties in adequately understanding the issue of distributions and reasoning with dynamic processes, reduces the scope of public initiatives in the field of health policies.

According to Krieger^14^, epidemiologists and other health professionals must disclose epidemiological theories about the distribution of diseases, harms, and health statuses, producing epidemiological knowledge that unveils population health and health inequalities, essential for multisectoral engagement in disease prevention, improvement of population health, achievement of equity in health, and advances in social justice.

Within the field of Collective Health, the tensions between the different sciences, disciplines, and knowledges are marked by fragmentation resulting from the proliferation of disciplines and exponential growth in the production of scientific knowledge in all *territories* and *villages* of the field, the predominance of technoscience based on the evaluation of technical effectiveness without adequate consideration of the effectiveness of interventions, and the deepening of inequalities between those who possess scientific and technical knowledge and those who do not.

Ayres^15^, discussing this fragmentation produced by the centrifugal movement of differentiation of areas within the field and the concern to preserve the identity of the field, problematizes the notions of territoriality and proposes the notion of villagization for the reconstruction of identity based on plurality. For him, the notion of villagization, adopted by the social movement of the native Brazilian peoples, refers to the meeting of different *tribes* in a single village, overcoming territories, ethnicities, languages, and cultures, breaking the barriers imposed by the territories, making borders permeable to exchanges and interactions, overcoming exclusions and preserving internal differentiations.

### Are dialogs still possible?

Fragmentation is not a prerogative of Collective Health. It is present in virtually all scientific fields and in the relations between different types of knowledge and learning from Science, Philosophy, and the Arts. The search for overcoming this fragmentation found in transdisciplinarity one of the possible alternatives for the integration of different knowledges.

In the First World Congress of Transdisciplinarity, transdisciplinarity was defined as that which crosses and surpasses discipline boundaries; new rationality open to another look at scientific objectivity; the reconciliation between Science, Philosophy, and the Arts; and understanding the multidimensionality of life^16^.

Maturana^17^ conceives transdisciplinarity as freedom of reflection to cross borders, freedom of expansion and understanding based on two assumptions: respect and detachment. He states that "going beyond the borders does not mean denying the former, eventually or completely abandoning a field, or even modifying it. Going beyond the border is an act of freedom"^17^.

In the field of Collective Health, Almeida Filho^18^ discussed transdisciplinarity by addressing the changes that occurred in scientific practice over time and the various epistemological attempts to overcome fragmentation and analytical procedures through approaches capable of synthesizing the constituents of complex objects, as conceived by Morin in the elucidation paradigm. For the French philosopher, elucidating means carrying out a provisional synthesis of multiple natural, historical, and social determinations, to deal with complex objects.

Among the various alternatives, Almeida Filho^18^ identifies transdisciplinarity as the one with greater capacity to overcome the paradigmatic crisis, as a cooperative production between different knowledges that take a strategic theme as a proposal for the investigation, starting to build an object of research in this process of interaction between types of knowledge, blurring and overcoming disciplinary boundaries, and also as a possibility of communication between agents in each subfield, established by the transit of subjects-agents of this crossing, in dialogue with the experts.

Resuming the topic in 2000, the author dealt with the relations between transdisciplinarity and intersectoriality in the field of Collective Health^19^. Here, transdisciplinarity appears as an overcoming strategy based on the production of synthetic and polysemous models resulting from the crossing of various disciplinary discourses.

While the transdisciplinary approach appears as an alternative in the production of knowledge of complex objects that constitute health, intersectoriality is presented as a strategy of solution to problems in the field of social practices. Collective Health is also constituted within the scope of practices that suffer the influence of power distribution in the health sector and in the political field in each of the social formations. The various fields of technological application within Collective Health demand intersectoral actions to the extent that they involve social actions within and outside the so-called health sector, considering that virtually all aspects of life have an impact on the health situation, whether in the production of the health status and well-being, in the protection against diseases and harms, or in the recovery of health^19^.

Luz^4^ states that the concept of field makes it easier to understand the multiplicity and co-occurrence of knowledges and practices in Collective Health, which makes the existence of a single discursive and practical paradigm in the field impossible. According to the author, there are three discursive models within the field that also translate into different intervention practices associated with the disciplinary nuclei that constitute the field. Historically, these models were organized in the classic Public Health, according to the paradigm of multidisciplinarity, later replaced by a paradigm of interdisciplinarity, which in Collective Health gave way to a paradigm of transdisciplinary. For Luz^4^, transdisciplinarity can be understood as the cooperative production between different types of knowledge based on a strategic theme for society, which in the process of intercommunication progressively develops the object of investigation.

In understanding the dynamics of scientific fields, several thinkers of the 20th century brought contributions that somehow sought to desacralize Science, considering the scientific work as a social practice, among others. In the first decades of the 20th century, Fleck^20^ drew attention to the peculiarities of medical and biomedical research that had as object the abnormal, the variety, rather than regularities that characterized the Exact Sciences, in addition to outcomes that should obtain immediate successes through intervention actions. For the author, scientific facts resulted from the articulation between what was perceived and the beliefs and conceptions of the collective of thought constituted by the practitioners of Science.

Bourdieu^21^, in his analyses of the scientific field, emphasized the possibility of overcoming the false hierarchies between basic research, strategic research, and technological research, pointing to the importance of considering possible inter-fertilization between different areas of knowledge and the development of common (transdisciplinary) objects for research.

Foucault^22^, in his *The Archaeology of Knowledge*, while recognizing and discussing the creation of regions delimited by disciplines and sciences, in the historical process that goes from discursive notions and practices to the formalization of theories, valued a more decentralized and less formalized set of diverse relationships originated in various social spheres, in the emergence and consolidation of new knowledges that often arise in the historical gaps that mark historical ruptures, without a definable synchronicity of historical becoming.

The idea of decentralization was resumed by Deleuze and Guattari^23^ associated with the notions of connection and heterogeneity, to address the networks between different types of knowledge and learning in a theory of the multiplicity of phenomena and manifestations of reality, breaking with more structural models.

These contributions, originated from different epistemological matrices, bring different subsidies that can guide the overcoming of the disciplinary perspective and the growing fragmentation between many types of knowledge. To this end, the organization of Science should abandon scientific disciplines as structuring units, promoting the reorganization around topics and practical-intellectual issues that may favor the encounter of different kinds of knowledge.

Such a change would require the revision and reorientation of methods and techniques so that they can adapt to the objects of investigation in a more elucidative and not only explanatory way, overcoming the understanding of the object (what) toward the why and how.

In this movement of change, decentralization and the rupture of false hierarchies between sciences are essential, as well as the rupture of disciplinary boundaries, so that, in fact, it is possible to construct other objects of investigation, in their complexity.

Among researchers and scientists, change will require greater cooperation, solidarity, and articulation to enable new forms of work (networks) and the reformulation of education procedures in graduate programs, in order to reconfigure thought styles^20^, *habitus* ^21^, and epistemes^22^.

## CONCLUSIONS

The challenges are not small, but the promise of the possibility of elucidating the complex issues that inhabit the field of Collective Health, requiring effective responses that, in addition to improving the health situation of the populations, may be guided by the moral imperative of reducing social inequalities and injustices, respecting human rights and building a more supportive and healthy society, seems a sufficient reward to motivate the search toward this new direction.

## Data Availability

does not apply.
