# Supplementary material for: Epidemiology in dialogues: belonging to the Collective Health field, practices and theoretical tensions
Source: Rev Bras Epidemiol. 2025 Nov 28;28(Suppl 1):e250002supl1. doi: 10.1590/1980-549720250002.supl.1 (PMC12667505; doi:10.1590/1980-549720250002.supl.1)
Supplement: Translation

# Translation: Epidemiologia em diálogos: pertencimento ao campo da saúde coletiva, práticas e tensionamentos teóricos

**DOI:** 10.1590/1980-549720250002.supl.1.2

**Authors:** Rita Barradas Barata

**Affiliations:** I Faculdade de Ciências Médicas da Santa Casa de São Paulo – São Paulo (SP), Brasil.

**Keywords:** Epidemiologia, Saúde pública, Filosofia

## Abstract

**Objetivo:**

Este ensaio trata do pertencimento da epidemiologia ao campo da saúde coletiva, discute teorias e contribuições da epidemiologia para o processo de trabalho em saúde e para a avaliação de impacto dos programas e ações de saúde coletiva, além dos tensionamentos práticos e teóricos na disciplina e no campo.

**Métodos:**

O ensaio foi construído com base na contribuição teórica de diferentes autores que pensaram e produziram reflexões sobre a epidemiologia ao longo do século XX e início do século XXI e nas experiências da autora. Ele está organizado em quatro blocos temáticos: o pertencimento da disciplina ao campo da saúde coletiva, as relações entre epidemiologia e práticas de saúde, as tensões práticas e teóricas no interior da própria disciplina e com as demais no próprio campo e a reflexão sobre os diálogos possíveis.

**Resultados:**

A autora sugere o recurso à transdisciplinaridade, a compreensão das dinâmicas dos campos científicos, a superação de falsas hierarquias entre os saberes, a superação dos limites disciplinares e o descentramento das conexões em rede como formas de enfrentamento e superação das dificuldades.

**Conclusão:**

A recompensa desse esforço está na possibilidade de alcançar respostas efetivas a problemas complexos que, para além de melhorar a situação de saúde das populações, possam estar orientados pelo imperativo moral de reduzir desigualdades e injustiças sociais respeitando os direitos humanos e construindo uma sociedade mais solidária e sadia.

## INTRODUÇÃO

A epidemiologia é uma das disciplinas nucleares do campo da saúde coletiva ligada à compreensão da dimensão populacional do processo saúde-doença tanto no estudo das distribuições dos estados e eventos de saúde quanto na elucidação das relações entre perfis epidemiológicos e determinantes sociais, culturais e políticos, em cada etapa do movimento histórico da sociedade.

Como disciplina dedicada à pesquisa estratégica no campo da saúde, os conhecimentos produzidos têm implicação nas práticas de prevenção e controle das doenças e agravos, manutenção do estado de saúde, formulação de estratégias de ação e avaliação dos impactos.

Os tensionamentos no interior da disciplina decorrem de diferentes perspectivas teóricas e metodológicas e, no interior do campo da saúde coletiva, da luta pelos capitais financeiros, políticos e simbólicos existente em qualquer campo científico.

Os objetivos do ensaio são discutir esses pontos e propor algumas formas de diálogo para a superação dos tensionamentos internos e externos.

## MÉtodos

Os argumentos apresentados têm origem nas reflexões da autora, com auxílio da seleção de livros e artigos de diferentes autores que pensaram a ciência e a epidemiologia ao longo do século XX e início do século XXI, para elaborar uma narrativa coerente e lógica abordando os diferentes aspectos indicados no título.

O ensaio contém quatro blocos: o pertencimento da epidemiologia ao campo da saúde coletiva, as relações entre a epidemiologia e as práticas de saúde, os tensionamentos teóricos e práticos no interior da própria disciplina e no campo da saúde coletiva, e os diálogos possíveis na própria disciplina e com outras no mesmo campo.

## RESULTADOS

### Pertencimento da epidemiologia ao campo da saúde coletiva

Segundo Alfredo Morabia^1^, a epidemiologia nasceu em 1662, na Inglaterra, quando John Graunt começou a analisar sistematicamente as listas de causas de óbito. As origens da disciplina dependiam da formulação do conceito de população e da comparação entre grupos, diferenciados por características demográficas, econômicas, sociais, culturais e políticas para o estudo das distribuições populacionais das doenças, agravos à saúde e estados de saúde e identificação dos determinantes dessas ocorrências.

Como o pensamento populacional não é algo intuitivo, a disciplina demorou a se constituir como tal, embora noções sobre saúde e higiene, bem como sobre doenças e medicina, fossem historicamente mais antigas.

As listas de mortalidade das paróquias de Londres, Inglaterra, foram um dos primeiros registros sistemáticos que permitiram a vigilância dos casos de peste para orientar as ações dos comitês de saúde em cada bairro. Em 1662, John Graunt publicou o livro *Natural and Political Observations Made Upon the Bills of Mortality*, com análise de dados dos 50 anos anteriores, constituindo um marco para o desenvolvimento dos conhecimentos epidemiológicos.

O conceito de saúde populacional emergiu apenas no fim do século XVIII, associado ao pensamento filosófico iluminista que adotava como princípio a ideia de que a proteção contra as doenças deveria ser estendida a todos os cidadãos e esperava que os avanços da ciência e da tecnologia permitissem prevenir as epidemias e melhorar a saúde da população^2^.

O desenvolvimento dos conhecimentos epidemiológicos e a constituição da disciplina científica se deram no século XIX, simultaneamente com as reformas de saúde pública nos países europeus e o movimento da medicina social. Desde o princípio, o movimento da saúde pública se viu em conflito com interesses econômicos dominantes revelando a ambivalência entre os benefícios que o crescimento econômico podia trazer para a saúde da população e a restrição às ações de saúde pública impostas pelas classes dominantes^2^.

Historicamente, todos os períodos de transição entre modos de produção apresentaram crescimento populacional acompanhado de maior suscetibilidade a doenças e piora relativa da saúde populacional. O acúmulo de riquezas por si só não garante os benefícios à saúde, sendo necessárias negociações sociais e políticas que orientem a repartição dos bens visando à maioria da população. Historicamente o crescimento econômico tem se mostrado intrinsecamente disruptivo, na ausência de políticas nacionais e locais que evitem ou minimizem os chamados quatro Ds: *disruption*, *deprivation*, *diseases* e *deaths*.

Nas últimas décadas do século XX, o processo de concentração das riquezas ocorreu de modo acelerado ao mesmo tempo que as políticas de bem-estar social implementadas nos países ricos e, em alguma medida, em países de renda média começaram a ser desmanteladas, aprofundando os efeitos negativos das desigualdades na distribuição das riquezas, sobre as condições de vida e saúde em todo o mundo.

A epidemiologia é a base para se compreender e alterar os padrões sociais de distribuição da saúde, doença e bem-estar, devendo produzir teorias para explicar os processos que moldam os perfis epidemiológicos na população, entender os determinantes das desigualdades sociais e orientar a elaboração de políticas públicas de enfrentamento. O primeiro dever de um epidemiologista é medir e tornar públicos os danos à saúde populacional resultantes do crescimento econômico e da concentração de riquezas, para mobilizar forças políticas e a opinião pública^3^.

Dependendo da teoria sobre o processo saúde-doença-cuidado, a compreensão acerca dos problemas será de cunho individual (teoria do estilo de vida, epidemiologia clínica) ou de cunho populacional (produção social da saúde e da doença), e consequentemente as propostas de intervenção serão distintas.

A saúde coletiva surgiu no Brasil como um movimento com raízes históricas na medicina social, em oposição à medicina preventiva e à saúde pública convencional. Conceituá-la como campo facilita a compreensão da multiplicidade e coexistência de saberes e práticas, constituídos em torno do objeto saúde-doença-cuidados na coletividade, como expressão do processo de organização social^4^.

O campo da saúde coletiva se estrutura em três eixos de saberes e práticas na dimensão coletiva: a epidemiologia, as ciências sociais em saúde e o planejamento, organização e gestão de políticas, programas e serviços de saúde^5^. Logo, mais do que pertencer ao campo da saúde coletiva, a epidemiologia é constituinte nuclear desse campo, juntamente com os outros saberes que o estruturam.

### Epidemiologia e atenção à saúde

Diferentes definições de epidemiologia, incluindo a do Dicionário de Epidemiologia^6^, denotam a dupla demanda pela produção de conhecimentos e aplicação deles na solução de problemas de saúde.

Para se compreender o papel da epidemiologia nas práticas em saúde, é importante considerar as particularidades do trabalho em saúde coletiva e sua finalidade primordial, que é modificar as condições populacionais de saúde^7,8^.

O objeto de trabalho em saúde é a *normatividade do modo de andar a vida* dos agrupamentos humanos. A capacidade essencial dos seres vivos mobilizada pela saúde é a capacidade de reconhecer os desafios cotidianos e responder a eles de forma normativa, criando novas normas diante das dificuldades^7,8^.

Os agentes e profissionais de saúde em seu trabalho operam saberes baseados nas ciências da vida e na prática profissional, combinando intuição, empatia, conhecimentos científicos e técnicas materiais e imateriais, desenvolvendo tecnologias e modelos de ação. Os arranjos tecnológicos articulam as necessidades sociais em saúde a certa racionalidade técnica (conhecimentos e instrumentos disponíveis) e formas de organização, visando à produção de um bem capaz de atender às necessidades em saúde^7,8^.

Os conhecimentos e as práticas epidemiológicas cumprem vários papéis nos arranjos tecnológicos em serviços de saúde orientados pela concepção da saúde coletiva e o enfoque populacional. Entre eles, podem ser citados a compreensão da distribuição da saúde, doença e bem-estar nos distintos grupos populacionais; a identificação dos processos que modelam essas distribuições; maneiras mais efetivas de enfrentar esses processos para impactar as distribuições no sentido da melhoria do estado de saúde; e avaliação das técnicas e tecnologias disponíveis e das intervenções^9^.

Particularmente, no que se refere à implementação e avaliação dos impactos dos programas e das políticas de saúde, a hipótese da equidade inversa desenvolvida por Cesar Victora e colaboradores amplia a compreensão sobre a capacidade de as intervenções em saúde coletiva reduzirem ou ampliarem as desigualdades sociais, dependendo do modelo de implementação adotado e da consideração prévia das desigualdades que modificam ou modulam os efeitos das intervenções^10^.

A hipótese da equidade inversa postula que novas intervenções atingem inicialmente as camadas sociais com nível socioeconômico mais alto e que, por sua condição de vida, compartilham situações que favorecem a incorporação das ações e serviços, e posteriormente essas inovações poderão ou não ser incorporadas pelas camadas mais pobres, dependendo do nível de cobertura populacional alcançado pelas intervenções^10^.

Dependendo ainda das estratégias de implementação usadas, novos programas e políticas poderão alcançar alta cobertura nas camadas que concentram poder e riqueza, com efeitos favoráveis concentrados no topo da pirâmide social e distanciamento em relação às outras camadas, ampliando as desigualdades. É possível também que propostas de alcance mais universal atinjam de maneira semelhante várias camadas sociais com diferentes posições de poder e riqueza, abandonando os mais pobres, com aumento da desigualdade desfavorável àqueles cuja inserção social é mais precária. Ou igualmente se podem identificar situações de um gradiente de coberturas inversamente proporcional à posição socioeconômica das diferentes frações de classe. Logo, é muito importante que a formulação das estratégias de implementação considere a existência das desigualdades e as características das diferentes frações sociais para evitar que as práticas em saúde sejam elas mesmas amplificadoras das desigualdades sociais em saúde^11^.

Desse modo, a epidemiologia, além de ser nuclear à estruturação do campo científico da saúde coletiva, apresenta-se como disciplina importante para as práticas em saúde, fornecendo conhecimentos relevantes para a organização do processo de trabalho em saúde na dimensão populacional, orientando a elaboração de programas e políticas baseados em evidências científicas e auxiliando na avaliação dos impactos das intervenções.

### Tensionamentos teóricos e práticos na disciplina e no campo

Os tensionamentos internos à própria disciplina vem sendo discutidos nos últimos 25 anos em torno da crise dos paradigmas explicativos, da pouca atenção dada às teorias de determinação social, da importância dada aos métodos e técnicas de análise voltados à elucidação etiológica em detrimento das análises relevantes para a práxis em saúde coletiva e o desenvolvimento incipiente do conceito de saúde e bem-estar^12–14^.

O empobrecimento teórico da disciplina e a predominância das questões de métodos e técnicas de análise têm consequências não apenas para o poder explicativo dos conhecimentos produzidos, mas também para as práticas no campo, visto que o enfoque na elucidação de mecanismos etiológicos tem enfraquecido a perspectiva da aplicação dos conhecimentos na solução dos problemas na dimensão populacional^12,14^.

Krieger^14^ destaca a permanência da oposição entre as teorias baseadas no chamado "estilo de vida" e as teorias da determinação social do processo saúde-doença. Aponta algumas tendências divergentes observadas no desenvolvimento da epidemiologia social: de um lado, a despolitização dos determinantes sociais em saúde; de outro, a ênfase renovada na economia política da saúde associada à discussão dos determinantes políticos partidários, comerciais e societários na saúde, ao aumento da visibilidade da saúde coletiva e da medicina social latino-americana, a teorias sobre saúde e direitos humanos, saúde populacional e outras.

Apesar dos múltiplos desenvolvimentos teóricos acerca da distribuição e dos determinantes da saúde e da doença, os autores assinalam a pouca atenção dada às questões de distribuição dos determinantes, das exposições e dos desfechos em saúde. Werneck^13^ destaca a extensa produção em epidemiologia no período 2001–2024 e "ausências gritantes" como: questões de gênero, racismo, populações em situação de vulnerabilidade social, população privada de liberdade, pessoas com deficiências, direitos humanos e saúde, preparação e resposta a emergências em saúde pública, crise climática, desinformação, interseccionalidade, entre outras. A essas ausências, podemos acrescentar ainda a polarização política e suas consequências na saúde e o novo obscurantismo e o crescente desprestígio social da ciência como processos histórico-sociais relevantes neste início do século XXI.

Todos esses tensionamentos, dilemas e lacunas teóricas têm reflexo no campo das práticas informadas pelo conhecimento epidemiológico. A escassez da perspectiva populacional e a pouca importância atribuída ao conhecimento e à compreensão das distribuições na organização dos serviços e no processo de trabalho em saúde reduzem a efetividade das políticas, programas e ações.

O despreparo dos profissionais de saúde, com escassa formação em epidemiologia e dificuldade para compreender adequadamente o problema das distribuições e para raciocinar com processos dinâmicos, reduz o alcance das iniciativas públicas no campo das políticas de saúde.

Segundo Krieger^14^, os epidemiologistas e outros profissionais de saúde têm o imperativo de tornar conscientes as teorias epidemiológicas sobre a distribuição das doenças, agravos e estados de saúde, produzindo conhecimentos epidemiológicos que desvelem a saúde populacional e as iniquidades em saúde, essenciais para o engajamento multissetorial na prevenção das doenças, melhoria da saúde populacional, alcance da equidade em saúde e avanços na justiça social.

No interior do campo da saúde coletiva, os tensionamentos entre as diferentes ciências, disciplinas e saberes são marcados pela fragmentação decorrente da proliferação de disciplinas e do crescimento exponencial na produção de conhecimentos científicos em todos os *territórios* e *aldeias* do campo, do predomínio da tecnociência baseada na avaliação da eficácia técnica sem a adequada consideração da efetividade das intervenções e do aprofundamento das desigualdades entre os detentores dos conhecimentos científicos e técnicos e os despossuídos de saberes.

Ayres^15^, discutindo essa fragmentação produzida pelo movimento centrífugo de diferenciação das áreas internas ao campo e a preocupação em preservar a identidade do campo, problematiza as noções de territorialidade e propõe a noção de aldeamento para a reconstrução da identidade com base na pluralidade. Para ele, a noção de aldeamento, adotada pelo movimento social dos povos originários brasileiros, remete à reunião de diferentes *tribos* em uma única aldeia, ultrapassando os territórios, etnias, línguas e culturas, rompendo as barreiras impostas pelos territórios, tornando as fronteiras permeáveis a trocas e interações, superando exclusões e preservando as diferenciações internas.

### Diálogos ainda são possíveis?

A fragmentação não é uma prerrogativa da saúde coletiva. Ela está presente em praticamente todos os campos científicos e nas relações entre diferentes conhecimentos e saberes oriundos da ciência, da filosofia e das artes. A busca pela superação dessa fragmentação encontrou na transdisciplinaridade uma das alternativas possíveis para a integração de diferentes saberes.

No primeiro Congresso Mundial de Transdisciplinaridade, a transdisciplinaridade foi definida como aquilo que atravessa e ultrapassa fronteiras disciplinares; nova racionalidade aberta a outro olhar sobre a objetividade científica; a reconciliação entre a ciência, filosofia e artes; e a compreensão da multidimensionalidade da vida^16^.

Maturana^17^ concebe a transdisciplinaridade como liberdade de reflexão para transpor fronteiras, liberdade de expansão e de compreensão baseada em dois pressupostos, o respeito e o desapego. Ele diz que "pisar além das fronteiras não significa negar a anterior, abandonar eventualmente ou completamente um campo, ou mesmo modificá-lo. Pisar além da fronteira é um ato de liberdade"^17^.

No campo da saúde coletiva, Almeida Filho^18^ discutiu a transdisciplinaridade discorrendo sobre as mudanças ocorridas no fazer científico ao longo do tempo e as diversas tentativas epistemológicas de superação da fragmentação e dos procedimentos analíticos mediante abordagens capazes de sintetizar os constituintes de objetos complexos, tal como concebido por Morin no paradigma de elucidação. Para o filósofo francês, elucidar significa realizar uma síntese provisória de múltiplas determinações naturais, históricas e sociais, para tratar de objetos complexos.

Entre as diversas alternativas, Almeida Filho^18^ identifica a transdisciplinaridade como aquela com maior capacidade de superação da crise paradigmática, como produção cooperativa entre distintos saberes que tomam um tema estratégico como proposta para a investigação, passando a construir um objeto de pesquisa nesse processo de interação entre saberes, borrando e superando os limites disciplinares, e ainda como possibilidade de comunicação entre agentes em cada subcampo, construída pelo trânsito de sujeitos-agentes desse atravessamento, em diálogo com os especialistas.

Retomando o tema em 2000, o autor tratou das relações entre a transdisciplinaridade e a intersetorialidade no campo da saúde coletiva^19^. Aqui a transdisciplinaridade aparece como estratégia de superação baseada na produção de modelos sintéticos e polissêmicos resultantes do cruzamento de diversos discursos disciplinares.

Enquanto a abordagem transdisciplinar aparece como alternativa na produção dos conhecimentos sobre objetos complexos que constituem a saúde, a intersetorialidade apresenta-se como estratégia de solução para problemas no campo das práticas sociais. A saúde coletiva também se constitui no âmbito de práticas que sofrem a influência da distribuição de poder no setor saúde e no campo político em cada uma das formações sociais. Os vários campos de aplicação tecnológica no interior da saúde coletiva demandam ações intersetoriais na medida em que envolvem ações sociais dentro e fora do chamado setor saúde, visto que praticamente todos os aspectos da vida têm incidência sobre a situação de saúde, seja na produção do estado de saúde e bem-estar, seja na proteção contra doenças e agravos, seja na recuperação da saúde^19^.

Luz^4^ afirma que o conceito de campo torna mais fáceis a compreensão da multiplicidade e a coexistência de saberes e práticas na saúde coletiva, o que torna impossível a existência no campo de um único paradigma discursivo e prático. Segundo a autora, há três modelos discursivos no interior do campo que também se traduzem em diferentes práticas de intervenção associados aos núcleos disciplinares que constituem o campo. Historicamente, esses modelos foram organizados na saúde pública clássica, conforme o paradigma da multidisciplinaridade, substituído posteriormente por um paradigma de interdisciplinaridade, que na saúde coletiva deu lugar a um paradigma transdisciplinar. Para Luz^4^, a transdisciplinaridade pode ser entendida como a produção cooperativa entre distintos saberes com base em um tema estratégico para a sociedade, que no processo de intercomunicação constrói progressivamente o objeto de investigação.

Na compreensão das dinâmicas dos campos científicos, diversos pensadores do século XX trouxeram contribuições que de certo modo buscaram dessacralizar a ciência, tratando o trabalho científico como uma prática social, entre outras. Nas primeiras décadas do século XX, Fleck^20^ chamava a atenção para as peculiaridades da pesquisa médica e biomédica que tinham como objeto o anormal, a variedade, em vez das regularidades que caracterizavam as ciências exatas, além de resultados que deveriam obter êxitos imediatos por meio das ações de intervenção. Para ele, os fatos científicos resultavam da articulação entre o percebido e as crenças e concepções do coletivo de pensamento constituído pelos praticantes da ciência.

Bourdieu^21^ enfatizou em suas análises sobre o campo científico a possibilidade de superar as falsas hierarquias entre pesquisa básica, pesquisa estratégica e pesquisa tecnológica apontando para a importância de se considerar a interfecundação possível entre diferentes áreas do conhecimento e a construção de objetos comuns (transdisciplinares) para a investigação.

Foucault^22^, em sua *A Arqueologia do Saber*, ao mesmo tempo que reconheceu e discutiu a criação de regiões delimitadas pelas disciplinas e ciências, no processo histórico que vai das noções e práticas discursivas até a formalização de teorias, valorizou um conjunto mais descentrado e menos formalizado de relações diversas originadas em vários âmbitos sociais, na emergência e consolidação de novos saberes que muitas vezes surgem nas brechas que marcam as rupturas históricas, sem uma sincronicidade demarcável no devir histórico.

A ideia de descentramento foi retomada por Deleuze e Guattari^23^ associada às noções de conexão e heterogeneidade, para falar das redes entre diferentes tipos de saberes e conhecimentos em uma teoria da multiplicidade dos fenômenos e manifestações da realidade, rompendo com modelos mais estruturais.

Essas contribuições, originadas de diferentes matrizes epistemológicas, trazem diferentes aportes que podem orientar a superação da perspectiva disciplinar e da fragmentação crescente entre muitos tipos de saberes. Para tanto, a organização da ciência precisaria abandonar as disciplinas científicas como unidades estruturantes, promovendo a reorganização em torno de temáticas e problemáticas prático-intelectuais que possam favorecer o encontro de diferentes saberes.

Tal mudança demandaria a revisão e reorientação dos métodos e técnicas para que possam se adequar aos objetos de investigação de forma mais elucidativa e não apenas explicativa, superando a compreensão sobre o objeto (o que) em direção aos porquês e ao como.

Nesse movimento de mudança, a descentralização e a quebra de falsas hierarquias entre as ciências são imprescindíveis, tanto quanto a ruptura das fronteiras disciplinares, para que de fato seja possível construir outros objetos de investigação, em sua complexidade.

Entre os pesquisadores e cientistas, a mudança exigirá maior cooperação, solidariedade e articulação para viabilizar novas formas de trabalho (redes) e a reformulação dos procedimentos formativos nos programas de pós-graduação, de modo a reconfigurar os estilos de pensamento^20^, os *habitus* ^21^, as epistemes^22^.

## CONCLUSÃO

Os desafios não são pequenos, mas a promessa da possibilidade de elucidação dos problemas complexos que povoam o campo da saúde coletiva, exigindo respostas efetivas que, para além de melhorar a situação de saúde das populações, possam estar orientadas pelo imperativo moral de reduzir as desigualdades e injustiças sociais, respeitando os direitos humanos e construindo uma sociedade mais solidária e sadia, parece recompensa suficiente para motivar a busca nessa nova direção.

## Data Availability

não se aplica.
